# Standard: Human gastric organoids

**DOI:** 10.1186/s13619-024-00218-6

**Published:** 2025-01-14

**Authors:** Fan Hong, Ronghui Tan, Ting Wang, Nanshan Zhong, Hongling Zhao, Rui-hua Xu, Lin Shen, Yingbin Liu, Xuebiao Yao, Dongxi Xiang, Lijian Hui, Jianping Xiong, Dong Gao, Bing Zhao, Zhifeng Miao, Jie Hao, Yong Li, Shijun Hu, Boqiang Fu, Guoqiang Hua, Lei Wang, Zhao-Lei Zeng, Chong Chen, Jianmin Wu, Changlin Wang, Chunnian Wang, Xianbao Zhan, Chen Song, Chunping Yu, Yingying Yang, Gengming Niu, Yalong Wang, Tongbiao Zhao, Ye-Guang Chen

**Affiliations:** 1 Guangzhou National Laboratory, Guangzhou, 510005 China; 2 The State Key Laboratory of Membrane Biology, Tsinghua‑Peking Center for Life Sciences, School of Life Sciences, Tsinghua University, Beijing, 100084 China; 3 The MOE Basic Research and Innovation Center for the Targeted Therapeutics of Solid Tumors, School of Basic Medical Sciences, Jiangxi Medical College, Nanchang University, Nanchang, 330031 China; 4 Beijing Institute for Stem Cell and Regenerative Medicine, Beijing, 100101 China; 5 State Key Laboratory of Oncology in South China, Collaborative Innovation Center for Cancer Medicine, Sun Yat-Sen University Cancer Center, Guangzhou, 510060 China; 6 Research Unit of Precision Diagnosis and Treatment for Gastrointestinal Cancer, Chinese Academy of Medical Sciences, Guangzhou, 510060 China; 7 State Key Laboratory of Holistic Integrative Management of Gastrointestinal Cancers, Beijing Key Laboratory of Carcinogenesis and Translational Research, Department of Gastrointestinal Oncology, Peking University Cancer Hospital and Institute, Beijing, 100142 China; 8 State Key Laboratory of Systems Medicine for Cancer, Shanghai Cancer Institute, Shanghai Jiaotong University School of Medicine, Shanghai, 200232 China; 9 Department of Biliary-Pancreatic Surgery, Renji Hospital Affiliated to Shanghai Jiaotong University School of Medicine, Shanghai, 200127 China; 10 MOE Key Laboratory of Cellular Dynamics, CAS Center for Excellence in Molecular Cell Science, University of Science and Technology of China, Hefei, 230027 China; 11 School of Life Science and Technology, Shanghai Tech University, Shanghai, 201210 China; 12 State Key Laboratory of Cell Biology, CAS Center for Excellence in Molecular Cell Science, Shanghai Institute of Biochemistry and Cell Biology, Chinese Academy of Sciences, University of Chinese Academy of Science, Shanghai, 200031 China; 13 University of Chinese Academy of Sciences, Beijing, 100049 China; 14 Department of Oncology, The First Affiliated Hospital of Nanchang University, Nanchang, 330031 Jiangxi China; 15 Jiangxi Key Laboratory for Individual Cancer Therapy, Nanchang, 330031 Jiangxi China; 16 Key Laboratory of Multi-Cell Systems, Shanghai Key Laboratory of Molecular Andrology, Center for Excellence in Molecular Cell Science, Shanghai Institute of Biochemistry and Cell Biology, Chinese Academy of Sciences, Shanghai, 200031 China; 17 Institute of Cancer Research, Shenzhen Bay Laboratory, Shenzhen, 518132 China; 18 Department of Surgical Oncology and General Surgery, The First Hospital of China Medical University, 155N Nanjing Street, Shenyang, 110001 Liaoning China; 19 Key Laboratory of Precision Diagnosis and Treatment of Gastrointestinal Tumors, Ministry of Education, China Medical University, Shenyang, 110001 Liaoning China; 20 Institute of Health Sciences, China Medical University, Shenyang, 110001 Liaoning China; 21 National Stem Cell Resource Center, Institute of Zoology, Chinese Academy of Sciences, Beijing, 100101 China; 22 State Key Laboratory of Stem Cell and Reproductive Biology, Institute for Stem Cell and Regeneration, Institute of Zoology, Chinese Academy of Sciences, Beijing, 100101 China; 23 Department of Gastrointestinal Surgery, Department of General Surgery, Guangdong Provincial People’s Hospital (Guangdong Academy of Medical Sciences), Southern Medical University, Guangzhou, 510080 China; 24 Department of Cardiovascular Surgery of the First Affiliated Hospital & Institute for Cardiovascular Science, Collaborative Innovation Center of Hematology, State Key Laboratory of Radiation Medicine and Protection, Suzhou Medical College, Soochow University, Suzhou, 510080 China; 25 National Institute of Metrology, Beijing, 100029 China; 26 Department of Radiation Oncology and Cancer Institute, Fudan University Shanghai Cancer Center Fudan University, Shanghai, 200433 China; 27 D1Med Technology (Shanghai) Inc, Shanghai, 201802 China; 28 Gastric Cancer Center and Laboratory of Gastric Cancer, State Key Laboratory of Biotherapy, West China Hospital, Sichuan University, Chengdu, 610041 Sichuan China; 29 Key Laboratory of Carcinogenesis and Translational Research (Ministry of Education), Center for Cancer Bioinformatics, Peking University Cancer Hospital and Institute, Beijing, 100142 China; 30 Peking University International Cancer Institute, Peking University, Beijing, 100191 China; 31 China National Institute of Standardization, Beijing, 100191 China; 32 Institute of Clinical Science, Zhongshan Hospital, Fudan University, Shanghai, 200433 China; 33 Department of Gastrointestinal Pathology, Ningbo Diagnostic Pathology Center, Ningbo, 315021 China; 34 Department of Oncology, Changhai Hospital, Second Military Medical University, No. 168 Changhai Road, Shanghai, 200433 China; 35 Huayi Regeneration Technology Co., Ltd, Chengdu, 611135 China; 36 Lilly (China) Research and Development Center, Shanghai, 201203 China; 37 Qilu Pharmaceutical Co., Ltd, Jinan, 250104 China; 38 Shang Hai OneTar Biomedicine Co., Ltd, Shanghai, 201203 China

**Keywords:** Standard, Human stomach, Gastric organoids, Terms, Definitions, Technical requirements, Labeling, Transportation, Storage

## Abstract

Organoid technology provides a transformative approach to understand human physiology and pathology, offering valuable insights for scientific research and therapeutic development. Human gastric organoids, in particular, have gained significant interest for applications in disease modeling, drug discovery, and studies of tissue regeneration and homeostasis. However, the lack of standardized quality control has limited their extensive clinical applications. The “Human Gastric Organoids” is part of a series of guidelines for human gastric organoids in China, which establishes comprehensive standards on terminology, technical specifications, testing methods, inspection rules, usage instructions, labeling, transportation, and storage, developed by experts from the Chinese Society for Cell Biology and its branch societies. Released on October 29, 2024, this guideline aims to establish standardized protocols, enhance institutional practices, and promote international standardization for clinical and research applications of human gastric organoids.

## Scope

This document specifies the ethical requirements, technical requirements, and testing methods, inspection rules, usage instructions, labeling, transportation, and storage for human gastric organoids.

This standard applies to the production and test of human gastric organoids derived from human gastric epithelial tissue and human pluripotent stem cells.

## Normative references

The following referenced documents are indispensable for the application of these documents. For dated references, only the edition cited applies. For undated references, the latest edition of the referenced document (including all amendments) applies.

*Pharmacopoeia of the People’s Republic of China* (2020 Edition, Part III).

## Terms and definitions

The following terms and definitions apply to this document.

### Organoids

Three-dimensional (3D) structures that grow from stem cells or progenitor cells in vitro, are capable of self-organization and renewal, consist of organ-specific cell types and can mimic the in vivo architecture and specific function of the original tissue (Clevers 2016; Fujii and Sato 2021; Kim et al. 2020; Wang et al. 2023).

### Human gastric organoids

Organoids that develop from human gastric stem cells of normal tissue or pluripotent stem cells, possess a variety of mature gastric epithelial cell types (Bartfeld et al. 2015; McCracken et al. 2017; McCracken et al. 2014).

### Organoid passage

Process of dissociating existing organoids into smaller fragments, or single cell via physical, chemical, or biological methods, and keeping them growing in vitro under the same culture conditions (Bartfeld et al. 2015).

### Organoid cryopreservation

Freezing process by which organoids are maintained at low temperature in an inactive state for maintaining cellular composition, gene expression, and functional properties.

### Organoid thawing

Process of bringing frozen organoids from an inactive to an actively growing state.

### Gastric stem cells

Cells that can self-renew and possess the ability to differentiate into all types of gastric epithelial cells (Barker et al. 2010; Kim and Shivdasani 2016; McCracken et al. 2017; Mills and Shivdasani 2011; Tan et al. 2020).

### Gastric stem cell differentiation

Process of gastric stem cells dividing into their daughter cells, including surface mucous cells, parietal cells, chief cells, mucous neck cells and enteroendocrine cells, et al. (McCracken et al. 2017; Wolffling et al. 2021).

### Gastric proliferative cells

Cells that are initially derived from gastric stem cells, characterized by high proliferative capacity and the ability to differentiate into various mature gastric epithelial cell types (Kim and Shivdasani 2016; Mills and Shivdasani 2011).

### Gastric surface mucous cells

Gastric epithelial cells located at the top of the gastric glands that secrete mucus, serving as the primary protective barrier of the gastric mucosa against stomach acid damage (Willet and Mills 2016).

### Gastric parietal cells

Cells in the neck region of the acid-secreting glands in the gastric body and antrum, specialized gastric epithelial cells abundant in tubulovesicles and mitochondria, that produce gastric acid and intrinsic factor, playing a crucial role in food digestion and the absorption of essential minerals such as phosphate, calcium, and iron (Miao et al. 2020; Yao and Smolka 2019; Yuan et al. 2017).

### Gastric chief cells

Cells located at the base of the acid-secreting glands in the gastric body and antrum are specialized gastric epithelial cells, rich in rough endoplasmic reticulum, zymogen granules, and mitochondria, and are primarily responsible for synthesizing and releasing pepsinogen (Bredemeyer et al. 2009; Goldenring et al. 2011; Ramsey et al. 2007).

### Gastric mucous neck cells

Cells typically located in the neck or base of the gastric glands, are specialized gastric epithelial cells responsible for secreting mucus, with cytoplasm filled with mucin granules, contributing to the gastric mucosal barrier (Bredemeyer et al. 2009; Ramsey et al. 2007).

### Gastric enteroendocrine cells

Gastric epithelial cells, with an irregular conical shape, typically dispersed singly among others, secrete gastric hormones in response to stimuli such as luminal food or pH changes, with cytoplasm containing numerous secretory granules, and can be further subdivided into distinct subtypes of gastric enteroendocrine cells, each with specific secretory functions (Busslinger et al. 2021; Li et al. 2014; Mitrovic et al. 2012).

## General requirements

### Raw materials

The acquisition of raw materials must comply with domestically recognized ethical standards and local laws.

Depending on the intended use, donor evaluation criteria should be established for the research and production of human gastric organoids.

### Process and information management

Critical factors influencing product quality during the procurement, preparation, testing, transportation, and storage of human gastric organoid raw materials should be documented, and a unique identification system should be implemented to ensure traceability throughout the process.

The minimum retention period for records must be clearly defined to ensure the integrity and security of documentation.

## Technical requirements

### Morphology

Human gastric organoids shall be cystic or bud-like, with a cavity in the middle and tightly contacting columnar epithelial cells on the outside under optical microscopy. The cavities and the edges of the junctions shall be clear, and the cells shall be transparent (Bartfeld et al. 2015).

Gastric organoids derived from human pluripotent stem cells should appear as vesicular or cystic under an optical microscope and possess glandular structures (McCracken et al. 2017).

### Chromosomal karyotype

The chromosomal karyotype of human gastric organoids shall be 46, XY or 46, XX (Bartfeld et al. 2015).

### Marker genes

Gastric organoids derived from human gastric corpus tissue and human pluripotent stem cells should express the marker genes *MKI67* for gastric proliferative cells, *MUC5AC* for gastric surface mucous cells, *ATP4B* for gastric parietal cells, *PGC* for gastric chief cells, *MUC6* for gastric mucous neck cells, and *CHGA* for gastric enteroendocrine cells. Additionally, gastric organoids derived from human gastric antrum tissue should also express the marker gene *LGR5* for gastric stem cells.

### Cell composition

Gastric organoids derived from human gastric corpus tissue and human pluripotent stem cells should contain MKI67-positive gastric proliferative cells, MUC5AC-positive gastric surface mucous cells, ATP4B-positive gastric parietal cells, PGC-positive gastric chief cells, MUC6-positive gastric mucous neck cells, and CHGA-positive gastric enteroendocrine cells. Additionally, gastric organoids derived from human gastric antrum tissue should also contain LGR5-positive gastric stem cells.

The proportion of gastric proliferative cells should be no less than 10%, gastric surface mucous cells no less than 5%, gastric chief cells no less than 5%, gastric mucous neck cells no less than 10%, and gastric enteroendocrine cells no less than 1%.

### Functional parameters

Human gastric organoids should possess the acid-secreting function of parietal cells and the pepsinogen-secreting function of chief cells (McCracken et al. 2017).

### Culture and growth

Human gastric organoids derived from healthy donors shall be able to be passaged for at least 2 generations in vitro after the initial culture.

Compared to the last generation, the passaged organoids shall have the same morphology, viability, marker gene expression, cell composition and other characteristics (Bartfeld et al. 2015).

### Organoids viability

After thawing, the number of viable gastric organoids should be no less than 50% of the number before cryopreservation, and the viable organoids shall be capable of being subcultured in vitro.

### Microorganisms

Organoids shall be negative for fungi, bacteria, mycoplasma, and virus.

### Identity

The identity of organoids shall match that of the donor tissue by STR analysis (Yang et al. 2024).

## Test methods

### Morphology

Observe organoid morphology by the inverted phase contrast microscope.

### Chromosomal karyotype

The method in “Preparation and quality control of animal cells for the production of biological products” from the *Pharmacopoeia of the People's Republic of China* (2020 Edition, Part III) shall be followed.

### Marker genes

The method can be found in Appendix A.

### Cell composition

The method can be found in Appendix B.

### Functional parameters

The acid-secreting function of parietal cells should be tested according to the method described in Appendix C.

The pepsinogen-secreting function of chief cells should be tested according to the method described in Appendix D.

### Culture and growth

Organoids cultured in vitro can be photographed using optical microscopy, with a scale bar for measuring their diameter and performing quantitative analysis.

### Organoids viability

Organoids viability shall be counted according to the method can be found in Appendix E.

### Microorganisms

#### Bacteria and Fungi

The “1101 Sterility Inspection Method” in *Pharmacopoeia of the People's Republic of China* (2020 Edition, Part III) shall be followed.

#### Mycoplasma

The “3301 Mycoplasma Inspection Method” in *Pharmacopoeia of the People's Republic of China* (2020 Edition, Part III) shall be followed.

#### Exogenous viral factors

The “3302 Exogenous Viral Factors Inspection Method” in *Pharmacopoeia of the People's Republic of China* (2020 Edition, Part III) shall be followed.

### STR

The method can be found in Appendix F.

## Instructions for use

The instructions should include at least the following information:

a) Organoid code;

b) Passage number;

c) Organoid number;

d) Production date;

e) Batch number;

f) Manufacturing organization;

g) Storage conditions;

h) Transportation conditions;

i) Contact information;

j) Usage instructions;

k) Standard reference number;

l) Production address;

m) Postal code;

n) Precautions.

Note: Endotoxin results should be provided upon user request.

## Labeling

The labels should include at least the following information:

- Organoid code;
- Passage number;
- Organoid number;
- Batch number;
- Manufacturing organization;
- Production date.

## Transportation and Storage

### Transportation

The transportation methods and conditions should be selected based on the requirements for the use of human gastric organoids to ensure their biological properties, safety, stability, and efficacy.

The transportation of human gastric organoids should consider, but not be limited to, factors such as the characteristics of the organoids, the container carrying the organoids, transportation route, conditions, equipment, methods, potential risks, and necessary safeguards.

The control measures for transportation conditions should include, but not be limited to, temperature range, vibration control, contamination prevention, equipment performance, and appropriate packaging.

Relevant inspection and technical guidance documents should be provided upon the user’s request.

The package should be checked during transportation, and if necessary, additional freezing sources (e.g., dry ice and liquid nitrogen) should be added to maintain the appropriate transportation temperature.

### Storage

Optimized cryopreservation protocols and methods should be employed to minimize damage to human gastric organoids during freezing and thawing processes, ensuring their normal functionality is minimally affected.

The cryopreservation information for human gastric organoids should be documented, including but not limited to:

- Organoid code;
- Batch number;
- Organoid number;
- Passage number;
- Freezing date;
- Cryoprotectant composition;
- Name of the operator.

The storage conditions for human gastric organoids should be documented, including but not limited to:

- Storage conditions;
- Storage date;
- Storage duration;
- Storage personnel.

## Data Availability

All data needed to evaluate the conclusions in the paper are present in the paper.

## Appendix A

### Cell Type Marker Gene Test (Real-time Fluorescence Quantitative PCR Method)

#### A.1 Instruments

*A.1.1* PCR-Cycler

*A.1.2* Real-time fluorescence quantitative PCR-Cycler

#### A.2 Reagents

Unless otherwise specified, the reagents used shall be analytically pure, and the water used for testing shall be deionized water.

*A.2.1* Phosphate Buffer Saline (PBS): pH 7.4

*A.2.2* Commercial RNA extraction kit

*A.2.3* Commercial RNA reverse transcription kit

*A.2.4* Commercial fluorescence quantitative PCR kit

*A.2.5* qPCR primers of GAPDH and target genes

#### A.3 Testing protocol

##### A.3.1 Organoid sample preparation

Aspirate the medium from the cultured organoids in vitro. Add an equal volume of PBS (A.2.1) and then aspirate. Repeat this step.

##### A.3.2 Organoid RNA extraction

Perform organoid RNA extraction using the commercial RNA extraction kit (A.2.2) according to the kit instructions.

##### A.3.3 Organoid cDNA preparation

One μg organoid RNA is used for organoid RNA reverse transcription using the PCR-Cycler (A.1.1) and the commercial RNA reverse transcription kit (A.2.3). Perform according to the kit and instrument instructions.

##### A.3.4 Gene expression determination

The organoid RNA reverse transcription product from step A.3.3 is used for gene expression determination. Perform real-time fluorescence quantitative test using the real-time fluorescence quantitative PCR-Cycler (A.1.2), the commercial fluorescence quantitative PCR kit (A.2.4) and related qPCR primers (A.2.5) according to the kit and instrument instructions. Determine the Ct value by referring to the detection curve, then obtain the expression value of *GAPDH* (*CtG*) and target genes (*CtM*).

##### A.3.5 Analysis of target gene expression

Taking *GAPDH* expression as a reference, obtain the expression level of target genes: X = *CtM*/*CtG*.

#### A.4 Calculation and analysis

Repeat the steps A.3.1 to A.3.5 for two more times. Calculate the expression levels of organoid target genes for three times, which are recorded as the average expression levels of organoid target genes.

#### A.5 Accuracy

The absolute difference of three independent measurements obtained under repeatability conditions shall not exceed 10% of the arithmetic mean.

## Appendix B

### Cell Composition Proportion Test (Immunofluorescence Staining Method)

#### B.1 Instruments

Laser confocal microscope.

#### B.2 Reagents

Unless otherwise specified, the reagents used shall be analytically pure, and the water used for testing shall be deionized water.

*B.2.1* PBS: pH 7.4

*B.2.2* Commercial immunofluorescence staining kit

*B.2.3* Antibodies of target proteins

#### B.3 Testing protocol

##### B.3.1 Organoid sample preparation

Aspirate the medium from the cultured organoids in vitro. Add an equal volume of PBS (B.2.1) and then aspirate. Repeat this step.

##### B.3.2 Organoid immunofluorescence staining

Perform organoid immunofluorescence staining using the commercial immunofluorescence staining kit (B.2.2) and antibodies of target proteins (B.2.3) according to the kit instructions.

##### B.3.3 Observation of immunofluorescence staining positive cells

The laser confocal microscope is used for observation and photography.

##### B.3.4 Analysis of target protein

Calculate the number of DAPI as the total number of cells in organoids (*M*). Calculate the number of cells with positive signal of target protein (*N*). The cell proportion with positive signal of target protein is obtained as *X = N/M*.

#### B.4 Calculation and Analysis

Calculate the cell proportion with positive signal of target protein in at least 30 organoids, and the average cell proportion is recorded as the proportion of target cell type in organoids.

## Appendix C

### Detection of Acid Secretion Function (Stimulating the Organoids with Histamine and Staining with Acridine Orange)

#### C.1 Instruments

Laser scanning confocal microscopy

#### C.2 Reagents

Unless otherwise specified, the reagents used shall be analytically pure, and the water used for testing shall be deionized water.

*C.2.1* PBS: pH 7.4

*C.2.2* Commercial histamine

*C.2.3* Commercial acridine orange

#### C.3 Testing protocol

##### C.3.1 Histamine Pretreatment of Organoids

For organoids cultured in vitro, replace the medium with histamine-containing medium (100 μM, from C.2.2) 30 minutes before staining. Incubate under normal culture conditions for 30 minutes.

##### **C.3.2 Acridine Orange Staining of Organoids**

Remove the culture medium from the in vitro cultured organoids. Add an equal volume of PBS (C.2.1) to wash the organoids. Repeat once. Stain the organoids with commercial 10 μM acridine orange (C.2.3).

##### **C.3.3 Observation of Positive Cells**

Use laser scanning confocal microscopy to observe and capture images at emission wavelengths of 500~550 nm and 600~650 nm.

#### C.4 Image Analysis

Analyze the images to calculate the ratio of positive cells at 600~650 nm emission wavelength to those at 500~550 nm emission wavelength in at least 30 organoids.

## Appendix D

### Detection of Pepsinogen Secretion Function (Enzyme-Linked Immunosorbent Assay (ELISA))

#### D.1 Instruments

Enzyme-linked immunosorbent assay (ELISA) plate reader

#### D.2 Reagents

Unless otherwise specified, the reagents used shall be analytically pure, and the water used for testing shall be deionized water.

*D.2.1* PBS: pH 7.4

*D.2.2* Commercial human gastric pepsinogen I enzyme-linked immunosorbent assay (ELISA) kit

#### D.3 Testing protocol

##### D.3.1 Organoid Retrieval

For organoids cultured in vitro, remove the culture medium. Add an equal volume of PBS (D.2.1) to wash the organoids. Remove the buffer.

##### D.3.2 Detection of Human Gastric Pepsinogen I

Perform the detection according to the instructions of the commercial human gastric pepsinogen I ELISA kit (D.2.2).

##### D.3.3 ELISA Plate Reader Detection

Measure absorbance using an ELISA plate reader.

#### D.4 Quantitative Analysis

Calculate the content of human gastric pepsinogen I based on the instructions provided in the commercial kit.

## Appendix E

### Organoids Viability (Calcein-AM Staining Method)

#### E.1 Instruments

*E.1.1* Inverted microscope.

*E.1.2* Fluorescence microscope

#### E.2 Reagents

Unless otherwise specified, the reagents used shall be analytically pure, and the water used for testing shall be deionized water.

*E.2.1* Dimethyl sulfoxide (DMSO) for cell culture.

*E.2.2* PBS: pH 7.4.

*E.2.3* Storage solution of Calcein-AM solution: 2 mmol/L in DMSO.

#### E.3 Testing protocol

##### E.3.1 Organoid observation in bright field

Place the organoids under the microscope to observe their morphology and status. Determine whether the organoid morphology meets the requirements of 6.1 by visual observation.

##### E.3.2 Organoid quantification

Add the Calcein-AM storage solution to the medium until the final concentration is 0.2 μmol/L, and incubate the mixture for 60 minutes at 37 ℃. Then clean the medium with Calcein-AM slowly with PBS (E.2.2) and add fresh medium. The organoids are observed and photographed by fluorescence microscope at 490 nm excitation wavelength and 515 nm emission wavelength. Living organoids are in green with clear edges. Count the number of organoids with a diameter ≥20 μm.

#### E.4 Accuracy

The absolute difference between the results of three independent determinations obtained under reproducible conditions shall not exceed 10% of the arithmetic mean.

## Appendix F

### Organoid Authentication by STR Profile

#### F.1 Instruments

*F.1.1* Centrifuge.

*F.1.2* PCR-Cycler.

*F.1.3* Electrophoresis apparatus.

*F.1.4* Microvolume UV Spectrophotometer.

#### F.2 Reagents

*F.2.1* Cell DNA extraction kit.

*F.2.2* STR DNA profiling kit.

#### F.3 Sample storage

The samples are prepared and stored below -80 ℃.

#### F.4 Testing protocol

##### F.4.1 Sample preparation

The organoids are cultured in the Matrigel to a stable growth state, and then mechanically pipetted out of the Matrigel. The mixture is collected in a centrifugal tube, the organoids are collected by centrifugation, and the supernatant is discarded.

##### F.4.2 Extraction of DNA

A) Perform genomic DNA extraction from organoids and primary tumor tissues according to the instructions of the Cell DNA extraction kit.
B) Measure the absorbance of extracted DNA by UV spectrophotometer to ensure that the ratio of A260/A280 is between 1.8 and 2.0.
C) DNA volume ≥ 20 μL, DNA concentration ≥ 50 ng/μL.

##### F.4.3 PCR amplification

A) Perform STR DNA amplification according to standard PCR amplification methods or the commercially approved kit instructions.
B) Use sterile water as the template for PCR amplification in the negative control group; use the DNA extracted from organoid and primary tumor tissue samples as templates in the sample detection group; use a DNA template with a confirmed STR profiling as the positive control group.
C) Detect the PCR products of three groups by agarose gel electrophoresis. Clear target band shall be observed in positive control but not in the negative control.

##### F.4.4 STR genotyping

Detect PCR products by capillary electrophoresis gene analyzer and STR genetic map data are obtained. The PCR banding pattern of organoids and primary tumor tissue shall be consistent.

#### F.5 Result analysis

*F.5.1* When STR alleles contain the same number of repeats, only one allele peak shall appear in the profile, when they contain different numbers of repeats, two allele peaks appear in the profile.

The test is considered valid when no allele peaks appeared in the negative control group and the positive control group is consistent with its standard genotyping data.

*F.5.2* If more than two allelic peaks are present at the STR locus of the tested sample, the sample shall be determined to be cross-contaminated after repeated experiments to exclude interfering factors such as mutations in the primer binding region, provided that the test is valid.

## Abbreviations

3D: Three Dimension
Ct: Cycle-threshold
DAPI: 4,6-Diamino-2-phenyl indole
DMSO: Dimethyl Sulfoxide
DNA: Deoxyribonucleic Acid
PBS: Phosphate Buffer Saline
PCR: Polymerase Chain Reaction
RNA: Ribonucleic Acid
STR: Short Tandem Repeat

## References

[CR1] Barker N, Huch M, Kujala P, van de Wetering M, Snippert HJ, van Es JH, et al. Lgr5(+ve) stem cells drive self-renewal in the stomach and build long-lived gastric units in vitro. Cell Stem Cell. 2010;6(1):25–36. 10.1016/j.stem.2009.11.013. 20085740 10.1016/j.stem.2009.11.013

[CR2] Bartfeld S, Bayram T, van de Wetering M, Huch M, Begthel H, Kujala P, et al. In vitro expansion of human gastric epithelial stem cells and their responses to bacterial infection. Gastroenterology. 2015;148(1):126–36 e6. 10.1053/j.gastro.2014.09.042. 10.1053/j.gastro.2014.09.042 PMC4274199 25307862

[CR3] Bredemeyer AJ, Geahlen JH, Weis VG, Huh WJ, Zinselmeyer BH, Srivatsan S, et al. The gastric epithelial progenitor cell niche and differentiation of the zymogenic (chief) cell lineage. Dev Biol. 2009;325(1):211–24. 10.1016/j.ydbio.2008.10.025. 19013146 10.1016/j.ydbio.2008.10.025 PMC2634829

[CR4] Busslinger GA, Weusten BLA, Bogte A, Begthel H, Brosens LAA, Clevers H. Human gastrointestinal epithelia of the esophagus, stomach, and duodenum resolved at single-cell resolution. Cell Rep. 2021;34(10): 108819. 10.1016/j.celrep.2021.108819. 33691112 10.1016/j.celrep.2021.108819

[CR5] Clevers H. Modeling Development and Disease with Organoids. Cell. 2016;165(7):1586–97. 10.1016/j.cell.2016.05.082. 27315476 10.1016/j.cell.2016.05.082

[CR6] Fujii M, Sato T. Somatic cell-derived organoids as prototypes of human epithelial tissues and diseases. Nat Mater. 2021;20(2):156–69. 10.1038/s41563-020-0754-0. 32807924 10.1038/s41563-020-0754-0

[CR7] Goldenring JR, Nam KT, Mills JC. The origin of pre-neoplastic metaplasia in the stomach: chief cells emerge from the Mist. Exp Cell Res. 2011;317(19):2759–64. 10.1016/j.yexcr.2011.08.017. 21907708 10.1016/j.yexcr.2011.08.017 PMC3210373

[CR8] Kim TH, Shivdasani RA. Stomach development, stem cells and disease. Development. 2016;143(4):554–65. 10.1242/dev.124891. 26884394 10.1242/dev.124891 PMC4760317

[CR9] Kim J, Koo BK, Knoblich JA. Human organoids: model systems for human biology and medicine. Nat Rev Mol Cell Biol. 2020;21(10):571–84. 10.1038/s41580-020-0259-3. 32636524 10.1038/s41580-020-0259-3 PMC7339799

[CR10] Li HJ, Johnston B, Aiello D, Caffrey DR, Giel-Moloney M, Rindi G, et al. Distinct cellular origins for serotonin-expressing and enterochromaffin-like cells in the gastric corpus. Gastroenterology. 2014;146(3):754–64 e3. 10.1053/j.gastro.2013.11.048. 10.1053/j.gastro.2013.11.048. 10.1053/j.gastro.2013.11.048 PMC3943955 24316261

[CR11] McCracken KW, Cata EM, Crawford CM, Sinagoga KL, Schumacher M, Rockich BE, et al. Modelling human development and disease in pluripotent stem-cell-derived gastric organoids. Nature. 2014;516(7531):400–4. 10.1038/nature13863. 25363776 10.1038/nature13863 PMC4270898

[CR12] McCracken KW, Aihara E, Martin B, Crawford CM, Broda T, Treguier J, et al. Wnt/β-catenin promotes gastric fundus specification in mice and humans. Nature. 2017;541(7636):182–7. 10.1038/nature21021. 28052057 10.1038/nature21021 PMC5526592

[CR13] Miao ZF, Adkins-Threats M, Burclaff JR, Osaki LH, Sun JX, Kefalov Y, et al. A Metformin-Responsive Metabolic Pathway Controls Distinct Steps in Gastric Progenitor Fate Decisions and Maturation. Cell Stem Cell. 2020;26(6):910–25 e6. 10.1016/j.stem.2020.03.006. 10.1016/j.stem.2020.03.006. 10.1016/j.stem.2020.03.006 PMC7275895 32243780

[CR14] Mills JC, Shivdasani RA. Gastric epithelial stem cells. Gastroenterology. 2011;140(2):412–24. 10.1053/j.gastro.2010.12.001. 21144849 10.1053/j.gastro.2010.12.001 PMC3708552

[CR15] Mitrovic O, Micic M, Radenkovic G, Vignjevic S, Ethikic D, Budec M, et al. Endocrine cells in human fetal corpus of stomach: appearance, distribution, and density. J Gastroenterol. 2012;47(11):1212–20. 10.1007/s00535-012-0597-9. 22544314 10.1007/s00535-012-0597-9

[CR16] Ramsey VG, Doherty JM, Chen CC, Stappenbeck TS, Konieczny SF, Mills JC. The maturation of mucus-secreting gastric epithelial progenitors into digestive-enzyme secreting zymogenic cells requires Mist1. Development. 2007;134(1):211–22. 10.1242/dev.02700. 17164426 10.1242/dev.02700

[CR17] Tan SH, Swathi Y, Tan S, Goh J, Seishima R, Murakami K, et al. AQP5 enriches for stem cells and cancer origins in the distal stomach. Nature. 2020;578(7795):437–43. 10.1038/s41586-020-1973-x. 32025032 10.1038/s41586-020-1973-x

[CR18] Wang Y, Lin H, Zhao L, Hong F, Hao J, Zhang Z, et al. Standard: Human intestinal organoids. Cell Regen. 2023;12(1):23. 10.1186/s13619-023-00168-5. 37314549 10.1186/s13619-023-00168-5 PMC10267020

[CR19] Willet SG, Mills JC. Stomach Organ and Cell Lineage Differentiation: from Embryogenesis to Adult Homeostasis. Cell Mol Gastroenterol Hepatol. 2016;2(5):546–59. 10.1016/j.jcmgh.2016.05.006. 27642625 10.1016/j.jcmgh.2016.05.006 PMC5025260

[CR20] Wolffling S, Daddi AA, Imai-Matsushima A, Fritsche K, Goosmann C, Traulsen J, et al. EGF and BMPs Govern Differentiation and Patterning in Human Gastric Glands. Gastroenterology. 2021;161(2):623–36 e16. 10.1053/j.gastro.2021.04.062. 10.1053/j.gastro.2021.04.062. 10.1053/j.gastro.2021.04.062 33957136

[CR21] Yang R, Qi Y, Kwan W, Du Y, Yan R, Zang L, et al. Paired organoids from primary gastric cancer and lymphatic metastasis are useful for personalized medicine. J Transl Med. 2024;22(1):754. 10.1186/s12967-024-05512-0. 39135062 10.1186/s12967-024-05512-0 PMC11318189

[CR22] Yao X, Smolka AJ. Gastric Parietal Cell Physiology and Helicobacter pylori-Induced Disease. Gastroenterology. 2019;156(8):2158–73. 10.1053/j.gastro.2019.02.036. 30831083 10.1053/j.gastro.2019.02.036 PMC6715393

[CR23] Yuan X, Yao PY, Jiang J, Zhang Y, Su Z, Yao W, et al. MST4 kinase phosphorylates ACAP4 protein to orchestrate apical membrane remodeling during gastric acid secretion. J Biol Chem. 2017;292(39):16174–87. 10.1074/jbc.M117.808212. 28808054 10.1074/jbc.M117.808212 PMC5625048

